# Temperature-Dependent Formation of Carbon Nanodomains in Silicon Oxycarbide Glass—A Reactive Force Field MD Study

**DOI:** 10.1021/acs.jpcc.4c05132

**Published:** 2024-12-16

**Authors:** Bernhard M. Kriesche, Felix R. S. Purtscher, Benedikt E. Hörfarter, Teja Stüwe, Victoria Greussing, Bettina Friedel, Engelbert Portenkirchner, Thomas S. Hofer

**Affiliations:** † Institute of General, Inorganic and Theoretical Chemistry Center for Chemistry and Biomedicine, University of Innsbruck, Innrain 80-82, A-6020 Innsbruck, Austria; ‡ Institute of Physical Chemistry, University of Innsbruck, Innrain 52c, A-6020 Innsbruck, Austria; § Department 4.5 Applied Radiometry, Physikalisch-Technische Bundesanstalt (PTB), Bundesallee 100, D-38116 Braunschweig, Germany

## Abstract

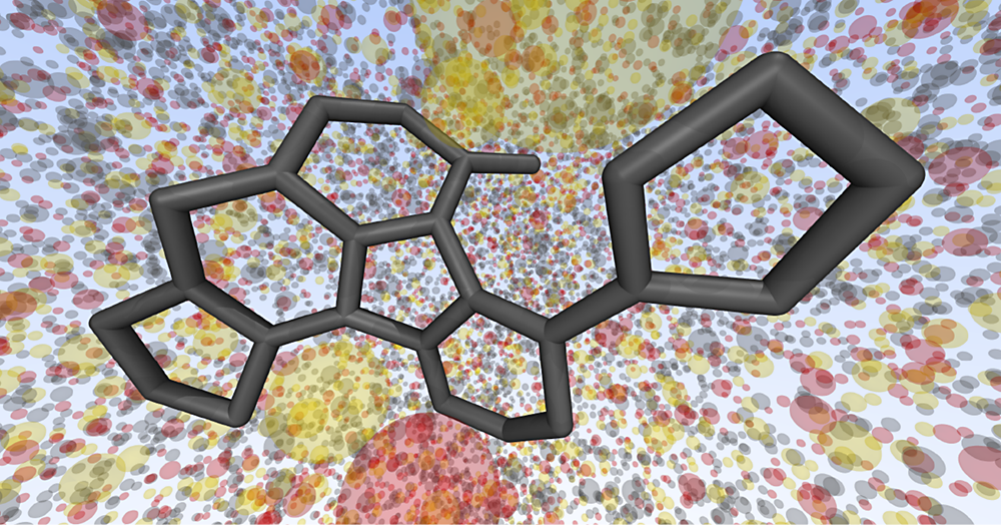

Novel anode materials for lithium-ion batteries (LIBs) are constantly being explored to further improve battery performance. In this work, ReaxFF molecular dynamics (MD) simulations are performed to model the early stages in the synthesis of nanostructured silicon carbide (SiC), which is one such promising material. The focus lies on its precursor, silicon oxycarbide glass of composition (Si_5_O_8_C_16_)_*x*_ (17 mol% Si, 28 mol% O, and 54 mol% C), in the following referred to as SiOC. The structure of the amorphous material is characterized via *NPT* MD simulations at temperatures ranging from 300 to 1000 K. To this end, a graph theoretical approach is employed to quantify the formation of segregated carbon nanodomains in the solid. Three algorithms for detecting nearest neighbors in the amorphous solid, a crucial prerequisite for the assembly of an atomic connectivity graph, are compared. It is shown that the temperature-dependent carbon aggregation follows an exponential trend, largely independent of the neighbor detection method. Also, the effects of variations in elemental composition are explored. Furthermore, the calculated powder X-ray diffraction patterns of the equilibrated silicon oxycarbide glasses are in good agreement with experimental measurements.

## 1 Introduction

Silicon carbide was first synthesized in large-scale in the 1890s via the Acheson process,^1^ by heating up a mixture of sand and coke, which provided the basis for industrial manufacturing. The process was improved and refined in the following decades leading to the advanced carbide furnaces present in industry today.^2^ Research interest in silicon carbide grew in the past decade as its properties facilitate applications in various technical disciplines.^3−5^ Due to its mechanical hardness and low cost, silicon carbide is used in manufacturing, e.g., in grinding and honing. Furthermore, the electrical properties enable applications in optics and microelectronic semiconductors, which have been extensively studied recently.^6−10^

The United Nations’ Sustainable Development Goal 7 (SDG 7) is outlined by the key principle *’Ensure access to affordable, reliable, sustainable and modern energy’*.^11^ One modern way to store and provide electrical energy are rechargeable lithium-ion batteries (LIBs), commonly used in a broad variety of portable electrical devices (e.g., phones, laptops, cameras) and electric cars.^12−14^ The success of LIBs can be explained by their high capacities and energy/power densities, their excellent charging/discharging properties as well as their comparatively low weight and toxicity.^14,15^ The widespread usage of these batteries has prompted continuous research efforts to enhance their electrochemical performance. In recent years, nanostructured silicon carbide (SiC) has been extensively studied both computationally and experimentally as a promising anode material for LIBs.^13−18^

These advances motivate this work to study SiOC, a precursor of silicon carbide, using computational methods. To this end, the experimental approach to be modeled herein is based on a sol–gel process to synthesize the amorphous precursor silicon oxycarbide glass upon annealing at elevated temperatures.^19−23^ It has been reported that the temperature employed in the annealing process has a critical influence on the microstructure of the resulting oxycarbide.^24^ In particular, the formation of segregated carbon and silica phases with an increasing degree of ordering has been observed upon an increase in annealing temperatures^25−27^ These carbon agglomerations have also been identified both experimentally by solid-state nuclear magnetic resonance (NMR)^28^ and computationally by *ab initio* MD simulations.^29^ Starting at approximately 1400 °C, the material transitions into a glass-ceramic state as these internal phases take on a partly crystalline character and the crystallization of β-SiC nanoparticles sets in. Upon further heating, carbothermal reduction occurs at the interface of the amorphous silica matrix and the segregated carbon phase to form SiC and gaseous CO.^27^ In addition to the applied duration and temperature in the annealing process, the elemental composition also has a critical influence on the characteristics of the resulting material. Depending on the desired product properties, silicon oxycarbide precursor materials of varying carbon content can be produced.^30−32^ Also, due to this microstructural variability and tunability of the properties via the composition, SiOC itself is considered a versatile material enabling a wide range of application possibilities.^24,33−36^ To obtain detailed insights into the associated chemical processes at the molecular level, this study investigates an amorphous SiOC model system with the reactive force field model ReaxFF^37^ and a previously determined parameter set^38^ in combination with an MD simulation setup. The microstructure and phase separation upon heating was studied to obtain an in-depth understanding of the carbon segregation process and compare the theoretical results to experimental findings, ultimately contributing to the development of improved synthesis procedures better tailored to individual applications.

## 2 Experimental Methods

### 2.1 SiOC Precursor Synthesis

The silicon oxycarbide glass precursor was synthesized via a sol–gel method as described by Friedel,^19^ via hydrolysis and condensation of tetraethoxysilane (TEOS). 54 g of TEOS (Thermo scientific, 98%) as silicon source and 67.6 g ethanol (VWR, >99.8%), were mixed together. In a second round-bottom flask 27.2 g sucrose (Sigma-Aldrich, >99.5%), as carbon source, was dissolved in 30 g deionized water (18.2 MΩ, Milli-Q, MILLIPORE) at 70 °C. After the solution was cooled to room temperature, 13.6 g of hydrochloric acid (Merck, 37%) was added as a catalyst. For the preparation of the sol, the two solutions were mixed together at room temperature under vigorous stirring. Following that, gelification to a lyogel took place in a sealed container at 60 °C overnight. The lyogel was dried to a black xerogel in a first step at 70 °C for 10 h and in a second step at 150 °C for another 5 h under evaporation of the solvent in an open container. Next, the precursor underwent pyrolysis of the sucrose at 450 °C followed by further sintering at 1000 °C for 3.5 h under argon gas flow. During sintering the silicate gel underwent vitrification, while the residual organic degradation products were carbonized.

### 2.2 Powder X-ray Diffraction (PXRD)

The PXRD patterns were recorded at room temperature with a Stoe Stadi P powder diffractometer in Debye–Scherrer geometry. The instrument was operated with Mo–Kα_1_ radiation (λ = 0.7093 Å, Ge(111) monochromator) and a Mythen 2 DCS4 detector. The obtained PXRD patterns were analyzed using the WinXPOW 3.07 software package.

### 2.3 X-ray Photoelectron Spectroscopy (XPS)

XPS analysis was conducted on a MultiLab 2000 (ThermoFisher Scientific), equipped with a monochromatic Al Kα_1_ X-ray source (1486.6 eV) and a hemispherical energy analyzer (Thermo Fisher Scientific, Alpha 110).

### 2.4 Raman Spectroscopy

To obtain Raman spectra of the sample a WITec WMT50 Raman microscope with a 532 nm laser was used. The spectra were recorded using a 1800 g mm^–1^ grating and a CCD camera.

## 3 Theoretical Methods

### 3.1 Molecular Dynamics Simulations

ReaxFF molecular dynamics (MD) simulations in the *NPT* ensemble at temperatures ranging from 300 to 1000 K and 1 atm were carried out using the Large-scale Atomic/Molecular Massively Parallel Simulator (LAMMPS, version 23Jun22)^39^ software package. The Nosé–Hoover thermostat^40−42^ and manostat^43,44^ algorithms were employed to achieve temperature and pressure control, with relaxation time τ_T_ = 0.1 ps and τ_P_ = 1.0 ps, respectively. A time step of 0.25 fs was used, as a larger time step potentially leads to instabilities during the MD simulation when employed in conjunction with reactive force fields.^45^ For charge calculations, the QEq scheme^46^ by Rappé and Goddard III, formulated by Nakano,^47^ was employed in every propagation step, with a tolerance of 10^–6^*e*, with *e* denoting the elementary charge. Moreover, neighbor lists store atoms within a radius of 12.0 Å and were updated every five MD steps. The ReaxFF parameter set developed by Newsome et al.^38^ for the investigation of silicon carbide oxidation processes was employed. Subject to temperature and initial configuration, the simulation time needed for the structural equlibration varied, so the MD simulations were continued until convergence of the carbon segregation process, monitored via the average carbon nanodomain (CND) size (*vide infra*), was observed.

### 3.2 Generation of Initial Configurations

Suitable SiOC starting structures were generated by randomly exchanging silicon and oxygen atoms for carbon in a 6 × 8 × 6 supercell (30.4 × 34.8 × 33.2 Å^3^) of crystalline silicon dioxide (SiO_2_, α-quartz) composed of 2592 atoms, taken from a previous research project. The probabilities for a given atom replacement were chosen based on the target composition. Naturally, this approach also achieves slight variations in composition upon repeated execution.

### 3.3 Carbon Nanodomain Size Analysis

To analyze the formation of differentiated carbon nanodomains in the SiOC precursor, the CND size in the solid is monitored over the course of the simulations. This approach, however, builds upon a suitable and robust definition of nearest neighbor (NN) contacts within the glass. As described in the following, three independent algorithms are applied for this purpose.

A straightforward method to detect NNs in an irregular solid is via the application of a *radial distance criterion*, i.e., to consider all atom pairs whose separation is less than the predefined cutoff *r*_c_ as neighboring. The value for the radial cutoff *r*_c_ is commonly selected based on radial pair distribution functions (RDF) *g*(*r*) and consequently this approach is in the following referred to as GC.^48^ The main disadvantage of this method, however, is that the parameter *r*_c_ is generally not transferable between systems and thus prior knowledge or preliminary analysis of the system of interest is required to determine an appropriate value for *r*_c_.

A different approach for neighbor identification is based on partitioning the computational domain among all atoms by means of a periodic *Laguerre–Voronoi tessellation*,^49,50^ which was performed with the Voronoi cell library *Voro++*(51) and is referred to as *LV* in the following. The Laguerre–Voronoi tessellation (also radical Voronoi tessellation) extends the regular Voronoi tessellation to include weights for different types of particles. In the context of atomistic calculations, atomic radii are a natural choice for these weighting factors — in particular the empirically determined radii as reported by Slater^52^ are used in this work. Trivially, atoms whose Laguerre cells share a face are considered nearest neighbors.

However, while Voronoi-based approaches can be applied in a straightforward way to perfectly ordered crystals, the tessellation tends to be unstable with respect to infinitesimal perturbations of the structure, e.g., due to finite temperature.^53,54^ Moreover, for inherently disordered particle systems, such as the amorphous material under investigation, Voronoi-like tessellations generally produce highly irregularly shaped cells with even rather distant particles sharing a small face.^55,53^ As a consequence of the considerable thermal motion of the atoms due to the comparatively high simulation temperature employed in this study this effect can be expected to be even more pronounced. This results in (i) the detection of a chemically unreasonably large number of nearest neighbors and (ii) large fluctuations in derived quantities over the course of the simulation. To address this issue, a threshold area for shared cell boundaries is introduced, that needs to be met for two atoms to be considered nearest neighbors. However, this scheme also requires careful parametrization, which is not chemically motivated and cannot be determined in an automated manner.

Since both NN detection algorithms presented so far involve (at least to a certain degree) arbitrary or intransferable parameters, a method less reliant on ad hoc interventions was sought. Accordingly, the third method, that finds application in this work is a modified Voronoi approach, in the following referred to as MV, that was developed by Malins et al. as part of their *Topological cluster classification (TCC)* algorithm.^49^ Conceptually, an unweighted, periodic Voronoi tessellation is performed and the resulting set of neighbors is further filtered, so that additionally the following two conditions are enforced^49^:

- The line connecting both particles passes through their Voronoi cells' shared face (*direct Voronoi neighbors*^49^).
- To permit a certain degree of asymmetry in four-membered rings, two particles on opposite sides of a tetragon are only considered neighbors if the angle they form with each of the remaining two particles as vertex is less than a set threshold π/3 < θ_thr_ ≤ π/2, equivalently expressed as the dimensionless *four-membered ring parameter f*_c_ ∈ (0.5, 1.0].^49^

Although thereby a parameter is also introduced in the MV algorithm, *f*_c_ is determined based on chemical reasoning and further it is not specific to the investigated system. To account for the amorphous nature and substantial thermal motion of the investigated systems, the setting permitting the highest degree of structural distortion is selected, i.e., *f*_c_ = 0.5 + ϵ (with ϵ = 2^–52^ ≈ 2.2 × 10^–16^ representing the machine precision of IEEE 64-bit floating point numbers). Further, to ameliorate the asymptotic complexity of the algorithm of  in the number of particles, a cutoff radius  is introduced to improve computational efficiency. However, given, that  is chosen large enough, e.g.,  is used in this work, it has no impact on the final result and its sole purpose is to exclude atom pairs at large distances early on in the algorithm.^49^

The bond information that the execution of all neighbor detection methods provides can be interpreted as an adjacency list and thus the specification of an undirected atomic connectivity graph. Based on the latter, a subgraph, consisting only of the carbon atoms is created by removing all vertices representing silicon and oxygen atoms, as well as edges involving them, while retaining the carbon–carbon connectivity information. Finally, the level of carbon aggregation by forming separated nanodomains, is determined by identifying the connected components of this subgraph via breadth-first traversal. This, in turn, facilitates the determination of the average number of carbon atoms constituting a nanodomain as
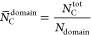
1with ,  and *N*_domain_ denoting the average number of carbon atoms per nanodomain, the total number of carbon atoms and the number of connected components/nanodomains, respectively.

By performing the described analysis for configurations drawn from consecutive simulation trajectories in regular intervals of 50 ps, the time evolution of the CND size is recorded. Once the structure in a given simulation is sufficiently converged, the analysis results from the last 5.0 ns are averaged to obtain a single average equilibrium value for a given state point and a standard deviation indicative of the extent of fluctuations in equilibrium.

### 3.4 Calculation of X-ray Diffractograms

Assessing the viability of the theoretical structure was done via comparison of the respective X-ray diffraction data of theoretical structures and their experimental equivalents. Theoretical PXRD patterns were calculated using the RIETAN-FP suite^56^ included in the VESTA program.^57^ Assuming ergodicity, time-averaged XRD patterns of the simulation trajectories were calculated, to compensate for the limited system size. To this effect, configurations were extracted in regular intervals from an equilibrated simulation trajectory and converted to the crystallographic information file (CIF) format (space group *P*_1_). These structures were then forwarded to VESTA/RIETAN-FP in an automated routine for the calculation of PXRD patterns, setting the radiation source to Mo–Kα_1_ radiation of wavelength λ = 0.70931 Å. To resemble the broadened shape of reflexes in experimental data, Gaussian-based weighted kernel density estimation^58^ with σ = 0.4° was applied to the individual reflexes generated by VESTA/RIETAN-FP. Finally, all generated PXRD patterns of a simulation trajectory were averaged to obtain a single ensemble-weighted pattern and then compared to the experimental measurement. To facilitate the comparison of the individual patterns a running average with 3°-wide window is added for both experimental and calculated data. In addition, all PXRD patterns are normalized according to the maximum intensity value of the running average in the range 2θ ≥ 25° and stacked along the *y*-axis.

## 4 Results and Discussion

### 4.1 Raman Spectroscopy

With respect to carbonaceous materials, Raman spectroscopy is often applied to investigate the carbon microstructure, *inter alia* based on the disorder-induced D band at approximately 1300–1400 cm^–1^ constituting a ring breathing mode, the G band arising from in-plane bond stretching of *sp*^2^ carbon at approximately 1580 cm^–1^ and the 2D band at approximately 2600–2700 cm^–1^, which represents an overtone of the D band and is also observed in defect free graphene. Further, along other features like peak broadness, the intensity ratio of D and G band, *I*_D_/*I*_G_, is employed as a semiquantitative measure for the degree of ordering.^59,60^ Accordingly, Raman spectroscopy has also been successfully used to characterize the free carbon phase in SiOC materials.^61,21,62,63^

A Raman spectrum was recorded as outlined in the Section 2 to characterize the microstructure of the free carbon phase of the SiOC sample sintered at 1000 °C (*cf.*Figure S19). Compared to published Raman spectra of samples treated at similar temperatures,^61,63^ the spectrum clearly shows the same characteristics, suggesting a similar microstructure of the carbon phase. In particular, the D and G bands are very broad and not well separated. Also, the second order region (2300–3300 cm^–1^) containing the 2D band shows elevated intensity but the individual bands are barely discernible. Furthermore, the D band shows a slightly larger intensity than the G band, *I*_D_/*I*_G_ > 1.

### 4.2 Generation of Initial Configurations

To obtain a faithful representation of the SiOC precursor used in the experimental procedures, consisting of approximately 17 mol% Si, 28 mol% O, and 54 mol% C, as determined via XPS, three independent initial configurations with slightly varying composition were generated via the mentionend procedure outlined in the Section 3. By altering the composition slightly from one randomization to the next, variations in the composition inherent to a macroscopic sample are taken into account in the simulations. Furthermore, one can assess how sensitive the nanodomain formation behavior is toward changes in composition.

The elemental compositions of the three generated random initial configurations (*RICs*) are summarized in Table 1.

**Table 1:** Number of Constituting Atoms of the Three Independently Generated SiOC Starting Configurations and the Experimental Composition for the Total Number of Atoms in the Simulation Cell, where *RICn* Indicates the Randomized Initial Configurations *n* = 1–3

system	*N*_Si_	*N*_O_	*N*_C_
*RIC1*	436	714	1442
*RIC2*	451	734	1407
*RIC3*	430	743	1419
exp	440	726	1400

### 4.3 Determination of Neighbor Detection Parameters

**Figure 1:**
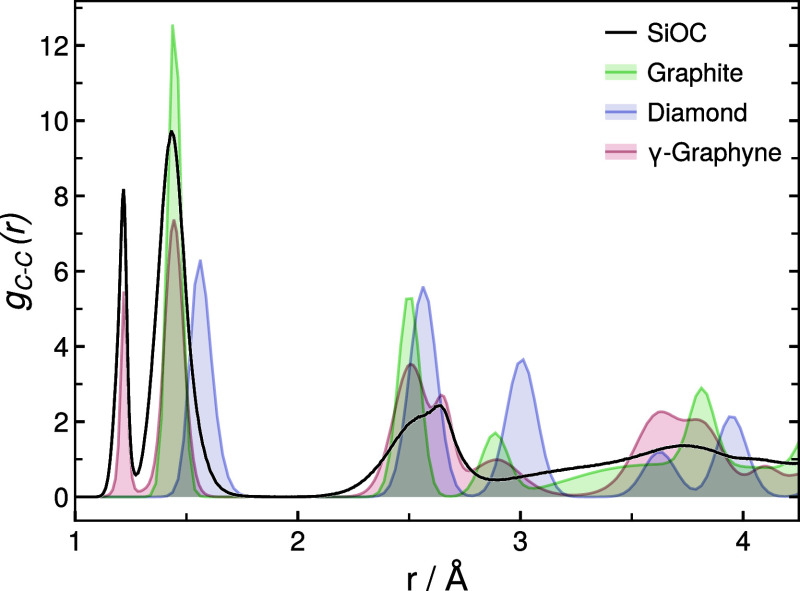
Carbon–carbon RDFs of an equilibrated SiOC simulation *RIC1* at 1000 K compared to reactive force field simulations of graphite, diamond, and γ-graphyne at the same statepoint for reference.

For the GC algorithm, the cutoff distance  is determined based on the carbon–carbon RDF *g*_C–C_, which was found to remain relatively constant across simulation temperatures. A typical carbon–carbon RDF *g*_C–C_ in SiOC is shown in Figure 1. C–C RDFs were also calculated for graphite, diamond and γ-graphyne based on short reactive force field *NPT* MD simulations at the same statepoint to obtain reference data for typical carbon–carbon nearest neighbor distances generated with the same interatomic potential (see Figure 1). These reference systems were chosen to reflect typically encountered carbon topologies, i.e., aromatic, single and triple carbon–carbon bonds as found in graphite, diamond and γ-graphyne, respectively. Based on the data in Figure 1, it can be concluded that all carbon atoms separated by significantly larger distances than approximately 1.8 Å can safely be considered not to be directly bonded (i.e., not nearest neighbors). Accordingly, the chosen cutoff distance of  Å — well within the broad minimum ranging from 1.8 to 2.1 Å — is an adequate choice.

**Figure 2:**
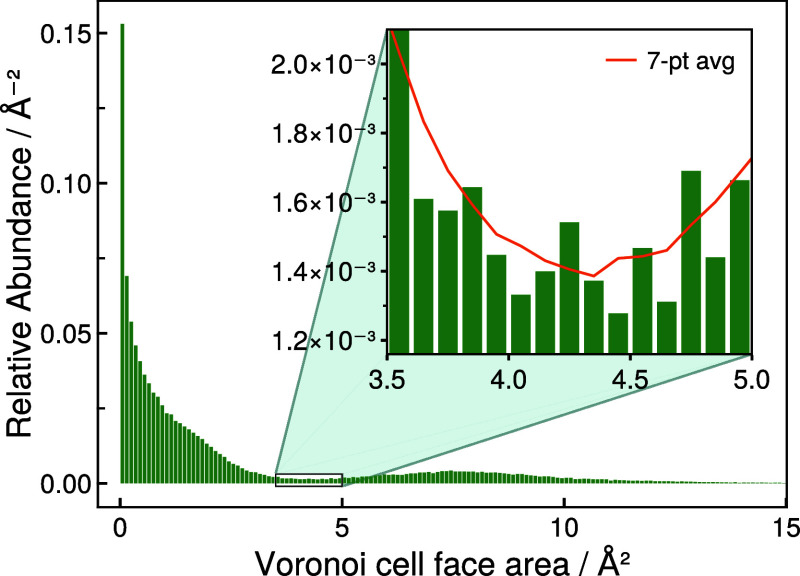
Normalized histogram of areas of shared carbon Voronoi cell faces.

For the LV algorithm, an appropriate face cutoff is determined by analyzing the distribution of shared face areas of adjacent Laguerre–Voronoi cells. For this purpose, Figure 2 presents a histogram showing the relative abundance of shared face areas of adjacent Laguerre–Voronoi cells representing carbon atoms over the course of multiple SiOC MD trajectories at temperatures from 300 to 1000 K. The distribution of face areas is clearly multimodal with a major fraction being very small, while the face areas that are useful for the classification of carbon–carbon bonds can be expected to be significantly larger. To provide a point of reference, the shared face area that connects the individual Voronoi cells within a perfectly symmetric graphite layer may be easily calculated to be approximately 7.5 Å^2^.

While the separation of overlapping distributions by a threshold value is always imperfect, one can identify a minimum in the histogram (see inset in Figure 2) at approximately 4.35 Å^2^, separating the small areas arising from the disordered nature of the system from the roughly unimodal distribution of areas centered at approximately 7.5 Å^2^. Subsequently, this minimum value of 4.35 Å^2^ was chosen as a reasonable threshold to separate artifacts introduced by the LV algorithm from data useful for carbon–carbon neighbor identification in SiOC.

Concerning the MV alogrithm, the cutoff radius is chosen as  Å, which is easily large enough to not exclude any potential nearest neighbors from the analysis, while still keeping the required computational ressources comparatively modest. The *four-membered ring parameter f*_c_ is furthermore set to the value permitting the highest degree of asymmetry of *f*_c_ = 0.5 + ϵ to account for the presence of highly distorted structures favored by the high simulation temperature.

### 4.4 Structural Investigations

**Figure 3:**
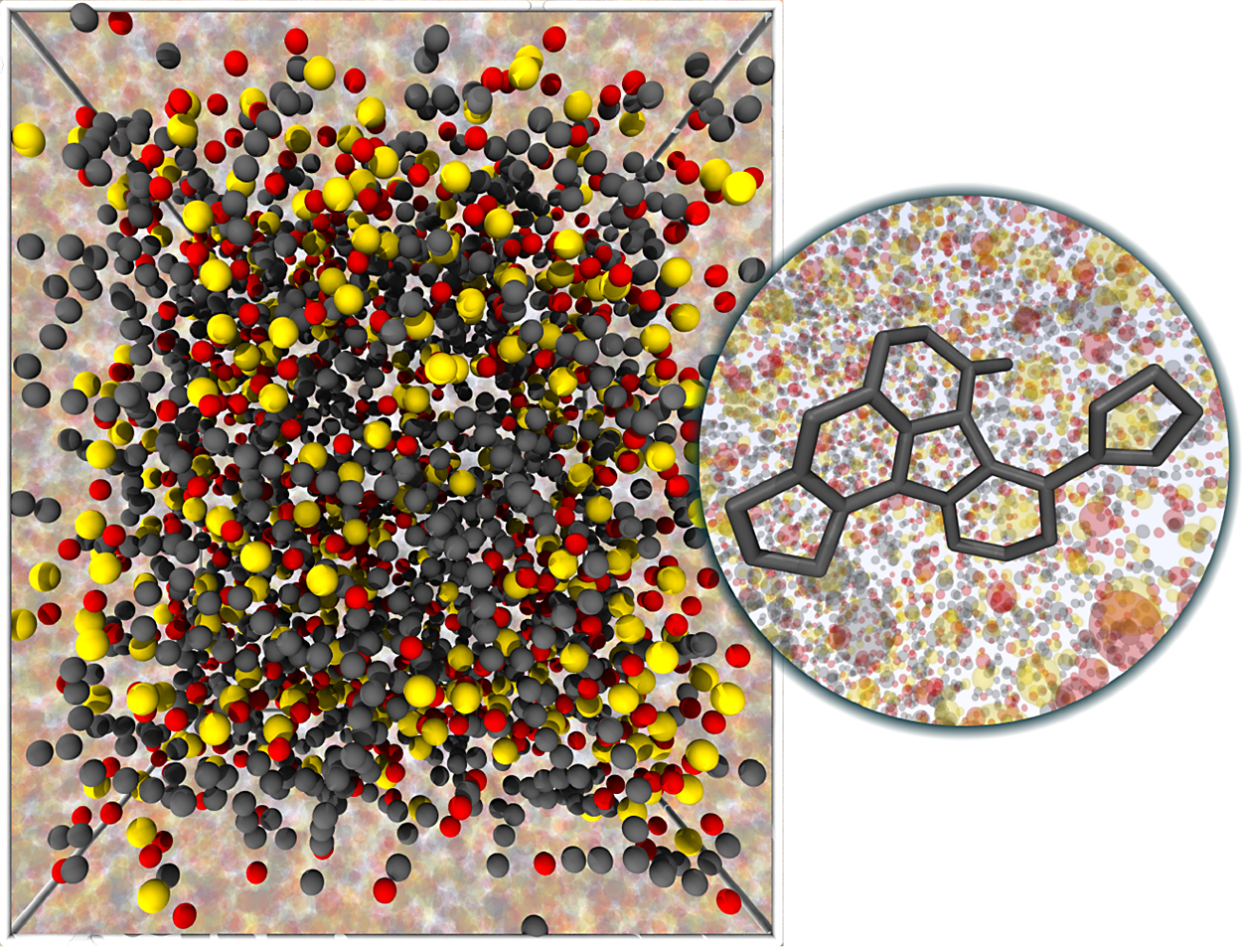
Equilibrated SiOC system *RIC2* at 1000 K with the inset demonstrating an emerging carbon layer.

The three randomly created SiOC initial configurations were equilibrated by ReaxFF MD simulations at temperatures 300, 400, 500, 600, 750, and 1000 K. During the course of the simulations an increasingly pronounced trend toward the formation of five- and six-membered carbon rings could be observed with increasing temperatures. At the highest investigated temperatures 750 and 1000 K, these carbon rings were not only ubiquitous, but further they combined to produce extended sheet-like carbon structures, which is in excellent agreement with the experimentally observed formation of polyaromatic carbon compounds in this temperature range reported previously.^25^Figure 3 presents a snapshot of such a carbon structure that formed in an SiOC system equilibrated at 1000 K.

**Figure 4:**
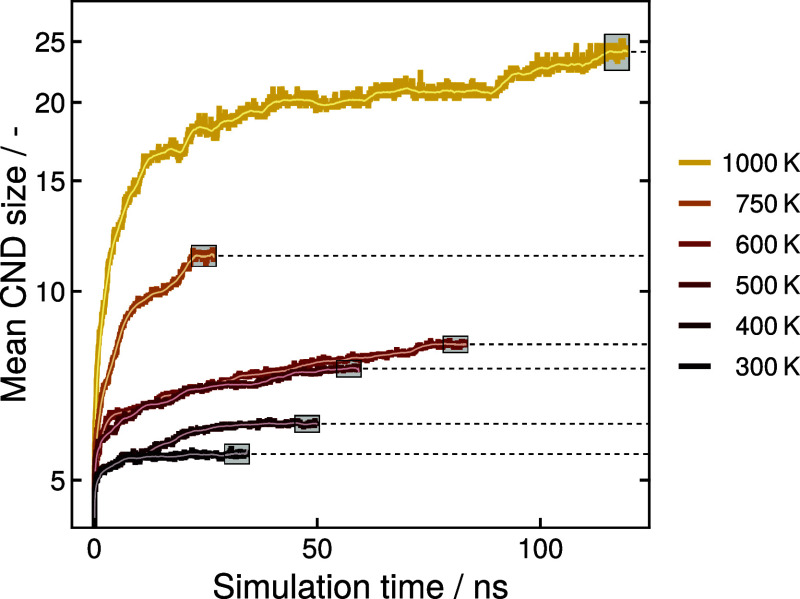
Time evolution of average CND size of system *RIC1* at various temperatures. The gray boxes indicate the terminal 5 ns of each simulation, and the resulting equilibrium values are indicated by the dashed horizontal lines.

Figure 4 shows the time evolution of the average CND size in the investigated SiOC system *RIC1* obtained with the MV^0.5^ algorithm at different temperatures. The time evolution of the CND size in all *RICn* systems, determined with all NN detection algorithms is contained in the Supporting Information (SI), Figures S1–S9. In general, the average CND size increases rapidly in the beginning, before converging slowly in an irregular, sometimes stepwise, manner. The simulation results show clearly that with higher temperatures, the carbon nanodomains tend to be larger, which often involves longer equilibration times.

**Figure 5:**
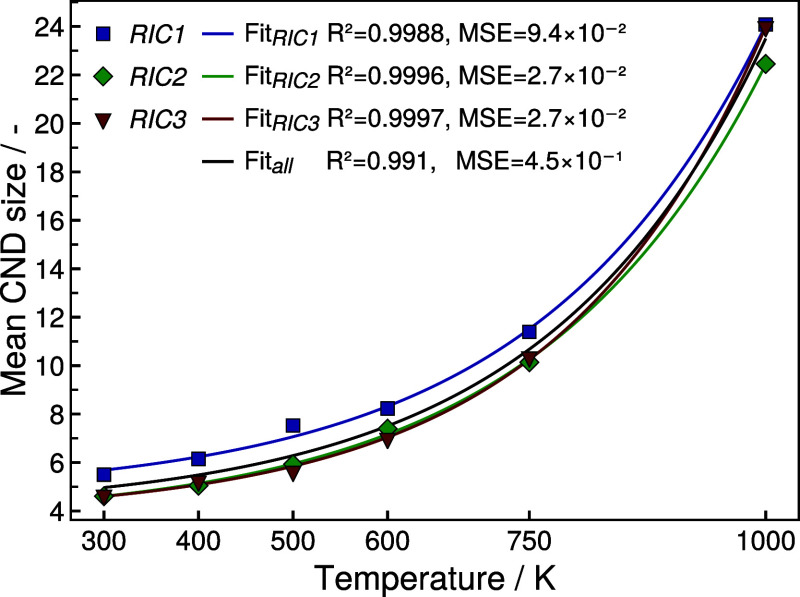
Equilibrium values of average CND size against simulation temperature determined with the MV^0.5^ algorithm and fit to an exponential model (see eq 2).

Subsequently, the terminal 5 ns of each equilibrated simulation were used to calculate a single average equilibrium carbon domain size (see Figure 4). The resulting temperature-dependent average CND sizes in SiOC are listed in Table 2. The three neighbor detection methods yield comparable results in the vast majority of cases, while the exact composition and randomized initial configuration seem to cause moderate fluctuations. Visualizing the obtained average equilibrium values of the CND size clearly reveals an exponential trend with respect to the applied simulation temperature (see Figure 5, Supporting Information Figures S10–S12).

**Table 2:** Ensemble Average of the Number of Carbon Atoms Per Nanodomain in SiOC in Equilibrium ± Standard Deviation w.r.t Simulation Time at Different Temperatures, Obtained with the GC^2.0^, LV^4.35^, and MV^0.5^ Neighbor Detection Algorithms

configuration	*T*/K	⟨*N*_C_⟩^GC^2.0^^	⟨*N*_C_⟩^LV ^4.35^^	⟨*N*_C_⟩^MV ^0.5^^
*RIC1*	300	5.389(11)	5.22(7)	5.50(5)
	400	6.112(07)	5.78(9)	6.15(4)
	500	7.466(29)	7.18(13)	7.53(5)
	600	8.201(23)	7.69(19)	8.23(6)
	750	11.53(7)	11.2(4)	11.39(13)
	1000	23.56(16)	25.2(13)	24.1(4)
*RIC2*	300	4.609(07)	4.60(05)	4.610(27)
	400	4.992(14)	4.88(06)	5.05(3)
	500	5.980(14)	5.95(10)	5.93(3)
	600	7.44(4)	7.11(17)	7.40(6)
	750	10.16(4)	9.74(26)	10.14(10)
	1000	22.41(22)	22.6(10)	22.45(26)
*RIC3*	300	4.606(06)	4.50(4)	4.642(27)
	400	5.277(14)	5.17(6)	5.23(4)
	500	5.643(16)	5.39(8)	5.63(4)
	600	7.038(28)	6.85(13)	6.99(4)
	750	10.441(25)	10.0 (3)	10.35(6)
	1000	23.61(16)	24.8(11)	24.0(4)

To capture the observed temperature dependence an exponential model is introduced,
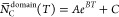
2with  and *T* denoting the number of carbon atoms per nanodomain and the temperature, respectively. Further eq 2 contains three parameters *A* and *C*, which are dimensionless, and *B* with the dimensionality of inverse temperature.

Based on eq 2, *A*, *B* and *C* can be determined for each combination of neighbor detection algorithm and initial configuration via nonlinear least-squares regression. The results of the regression procedure including *R*^2^ and MSE as measures for the quality of the fit are listed in Table 3 and the obtained exponential model is also included in Figure 5 and Supporting Information Figures S10–S12. First, it can be noted, that the chosen exponential model is able to capture the temperature-dependent behavior extremely well for all SiOC configurations *RICn* individually, independent of the NN algorithm employed. The exponential fit to the data from all SiOC configurations combined is significantly worse, with the MSE being almost an order of magnitude larger compared to the individual fit results. This is a clear indication, that the introduced variations in elemental composition have a noteworthy effect on the carbon domain size. This is also reflected by the fact, that the determined values for the parameter *A*, and to a lesser degree also for *B* and *C*, deviate notably across the employed initial configurations *RICn*. To this effect, the carbon segregation behavior seems to be very sensitive to compositional changes.

**Table 3:** Resulting Parameters of the Exponential Model (see eq 2) for the CND Size in the SiOC/*RICn* (*n* = 1–3) Systems Individually and Combined, Using Data Obtained with the GC^2.0^, LV^4.35^, and MV^0.5^ Neighbor Detection Algorithms

algorithm	data set	*A*/–	*B*/*K*^–1^	*C*/–	*R*^2^/–	MSE/–
GC^2.0^	*RIC1*	0.4028	0.003868	4.263	0.9989	0.078
	*RIC2*	0.3179	0.004084	3.510	0.9995	0.035
	*RIC3*	0.2493	0.004383	3.652	0.9995	0.037
	combined	0.3154	0.004117	3.825	0.992	0.38
LV^4.35^	*RIC1*	0.2432	0.004446	4.435	0.9987	0.115
	*RIC2*	0.2075	0.004503	3.819	0.9994	0.043
	*RIC3*	0.1568	0.004895	3.843	0.9995	0.041
	combined	0.1985	0.004619	4.043	0.990	0.54
MV^0.5^	*RIC1*	0.3078	0.004146	4.619	0.9988	0.094
	*RIC2*	0.3003	0.004141	3.567	0.9996	0.027
	*RIC3*	0.2141	0.004548	3.764	0.9997	0.027
	combined	0.2687	0.004285	3.996	0.991	0.45

In contrast, the results obtained by the three different NN detection algorithms are in general consistent. In particular for parameters *B* and *C* the agreement across NN algorithms (for the same *RICn*) is astonishing, considering the fundamentally different approaches of the individual algorithms. For parameter *A*, however, the deviation across NN algorithms is approximately of the same magnitude as the differences across the *RICn* systems.

**Figure 6:**
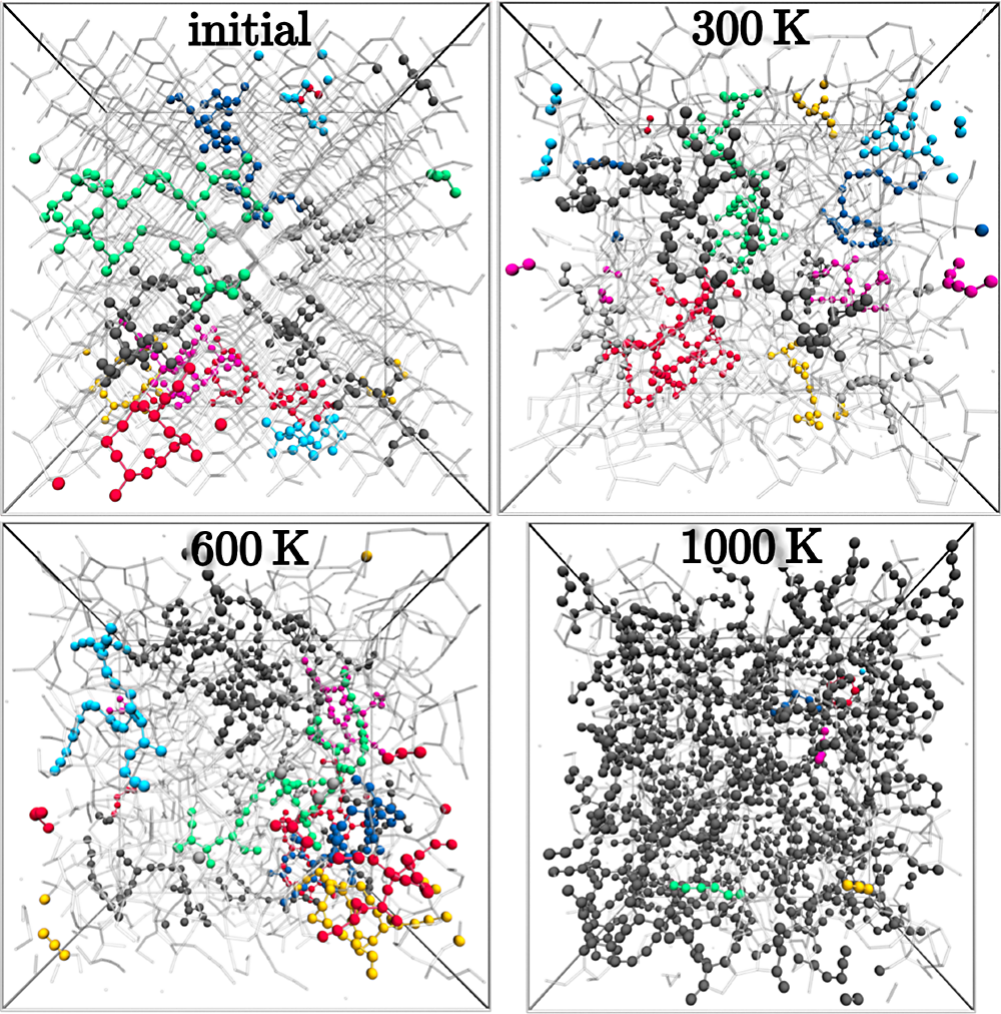
Snapshots of the SiOC/*RIC1* system at different simulation temperatures. All atoms are indicated by semitransparent sticks. The six largest carbon domains are additionally highlighted in different colors. Due to the anisotropic pressure coupling, the simulation cell sizes differ.

Further, at the highest investigated temperature, 1000 K, the single largest carbon domain contains already >90% of the available carbon atoms in the system, which is demonstrated in Figure 6. This also indicates, that for the investigation of the carbon segregation behavior in SiOC at temperatures >1000 K the simulation systems need to be increased drastically in size to enable an adequate representation.

**Figure 7:**
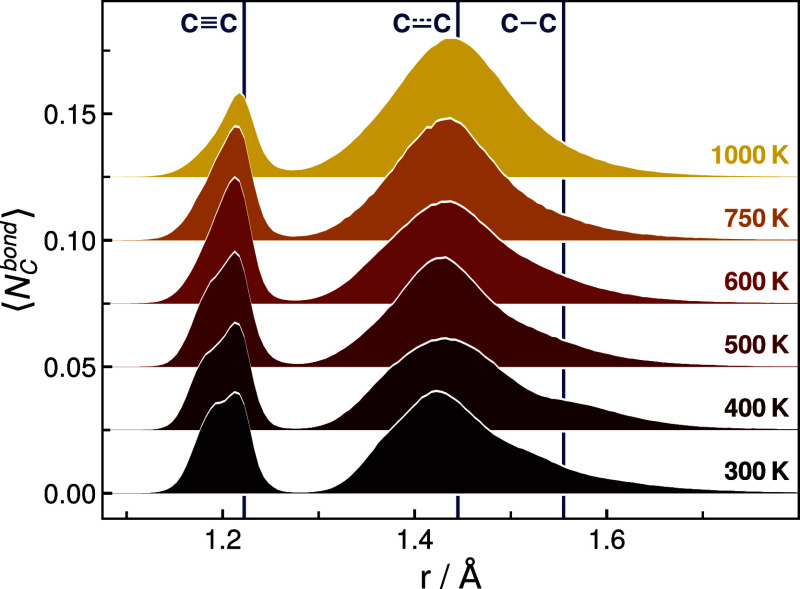
Time-averaged histogram of CC distances in SiOC/*RIC1* after equilibration at different temperatures, normalized according to the total number of carbon atoms in the system. Reference values indicated via C–C, C=C, and C≡C denote the median values of single, aromatic, and triple carbon–carbon bond distances extracted from ReaxFF MD simulations of diamond, graphite, and γ-graphyne, respectively.

The distribution of carbon–carbon bond distances in SiOC/*RIC1* presented in Figure 7 for temperatures 300–1000 K further illustrates the observed carbon segregation process and the formation of a free carbon phase (see Supporting Information, Figures S13–S15 for the corresponding histograms of all investigated systems). In particular, the number of carbon–carbon distances in the range found in aromatic carbon compounds increases notably at 750 and 1000 K compared to the lower temperatures. Also, this finding is in full agreement with the reported formation of polyaromatic carbon species in SiOC at temperatures 600 K < *T* ≤ 1000 K.^25^ Simultaneously, the abundance of carbon–carbon distances as commonly found in triple bonds decreases. Finally, it seems worth mentioning that the number of carbon–carbon distances corresponding to aliphatic carbon species appears to be independent of the simulation temperature.

**Figure 8:**
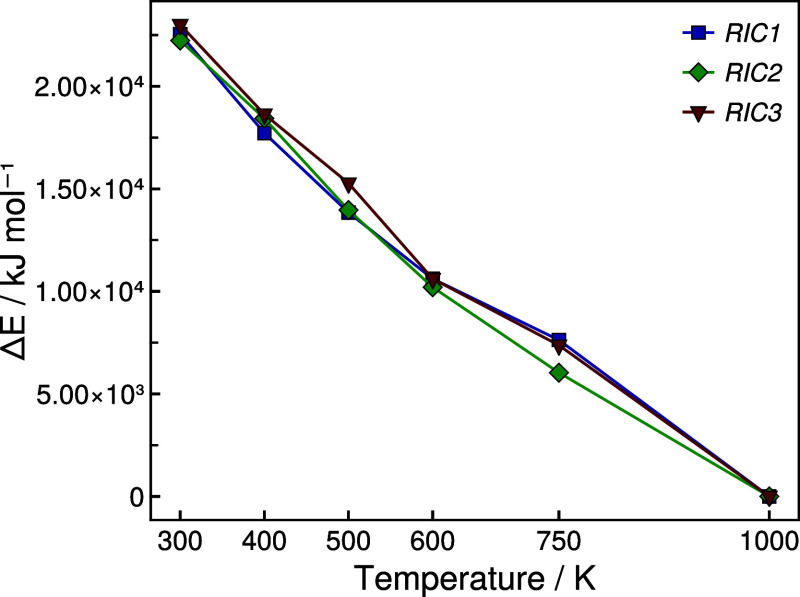
Total energy of the different SiOC/*RICn* (*n* = 1–3) systems equilibrated at different temperatures after subsequent cooling to 300 K, calculated as Δ*E*(*T*) = *E*(*T*) – *E*(1000 K).

After successful equilibration at the respective target temperature, each simulation was again cooled to 300 K which did not alter the average CND size. Subsequently, the energy difference relative to the system lowest in energy, was plotted against the equilibration temperature (see Figure 8) which indicates, that the nanodomain formation in SiOC is a strongly exothermic process. Furthermore, the strong correlation of energy and equilibration temperature suggests that the carbon segregation process is subject to kinetic control.

### 4.5 Powder X-ray Diffraction Patterns

All SiOC structures equilibrated at elevated temperatures were cooled to 300 K at 1 atm of pressure and sampled for 25 ps. Next, time-averaged PXRD patterns of the individual systems were generated as outlined above. Considering that the structural order in the investigated material is certainly not as pronounced as in crystalline systems, the recorded diffraction patterns are expected to show broad distributions, rather than sharp reflexes. Accordingly, the goal of the calculation of time-averaged XRD patterns was to investigate whether similar distributions can be detected in the MD data.

**Figure 9:**
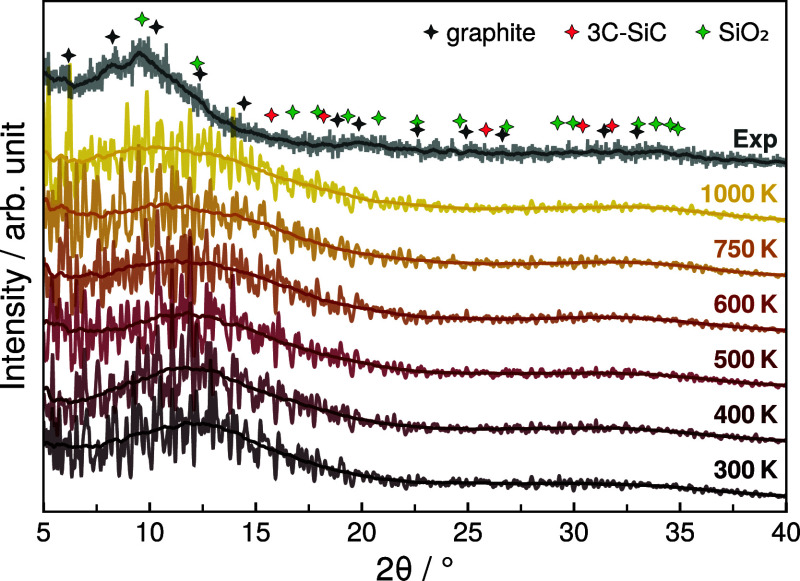
Time-averaged PXRD patterns of SiOC/*RIC2* at different equilibration temperatures compared to the experimentally measured diffractogram. Reflexes obtained for graphite, 3C-SiC, and SiO_2_ at the respective minimum configuration with intensities being ≥1.0% of the main reflex are indicated as diamond-shaped markers.

The computationally predicted PXRD pattern of *RIC2* are shown in Figure 9, including an experimentally measured diffractogram of silicon oxycarbide glass. Additionally, the PXRD patterns of graphite, 3C-SiC and SiO_2_ were calculated from their minimum energy geometries and all reflexes at angles 5° ≤ 2θ ≤ 35° with intensities of at least 1.0% of the strongest reflex are indicated in Figure 9. The PXRD patterns of all investigated SiOC/*RICn* systems and a list of the marked reflexes of graphite, 3C-SiC and SiO_2_ can be found in the Supporting Information, Figures S16–S18 and Table S1, respectively.

With regards to the experimental data, the recorded PXRD pattern lacks sharp reflexes, confirming the predominantly amorphous nature of the material. Furthermore, the PXRD pattern is dominated by multiple broadened peaks in the range 2θ < 15°, which largely coincide with the calculated reflexes of graphite at 6.18, 8.25, 10.31 and 12.38° and SiO_2_ at 9.64 and 12.24°. This reflects the fact that, in addition to the generally amorphous structure in the solid, small crystalline graphite and SiO_2_ domains are present. This is consistent with previous results that suggest approximately 1000 °C as the threshold of the initial formation of these increasingly ordered domains.^26^ Also, the absence of peaks clearly identifiable as SiC reflexes in the experimental pattern aligns with the suggested higher threshold temperature for initial SiC formation of approximately 1400 °C.^27^

In comparison to the experimental PXRD pattern, the calculated patterns show a similar trend dominated by multiple peaks at lower angles. Notably, in the range of 25° ≤ 2θ ≤ 35° the calculated XRD patterns are virtually identical to the experimental one, while particularly for smaller angles, the observed divergence increases. According to the Bragg equation^64^ at the respective measurement wavelength (0.7093 Å), angles in the range of 25° ≤ 2θ ≤ 35° correspond to distances 1.18 Å ≤ *d* ≤ 1.64 Å, assuming first order reflection. Consequently, this part of the XRD pattern can be regarded as dominated by short-range order including typical bond lengths encountered in the investigated system. The part of the XRD pattern at smaller angles 2θ < 25° is increasingly dominated by the long-range order of the system. Accordingly, this suggests that the short-range order in the experimental material is captured very well by the simulations conducted in this work. The fact that the part of the XRD pattern at smaller angles is only qualitatively captured by the simulation results is likely due to a number of reasons.

Most importantly, in agreement with published literature,^25^ our results indicate that at 1000 K — the highest simulation temperature used in the theoretical parts of this work — the long-range order is not yet as pronounced with the segregated carbon phase primarily consisting of polyaromatic ring systems. Also, the limited system size accessible to computer simulation can be expected to alter the representation of long-range effects, particularly for amorphous systems. Furthermore, deviations between the lattice parameters of the experimental and simulated SiOC systems might also partly account for the observed differences in the PXRD patterns at smaller angles. Irrespective of these considerations, the predicted PXRD patterns reflect the experimental measurements very well.

## 5 Conclusions

In the work presented here, silicon oxycarbide glass with composition (Si_5_O_8_C_16_)_x_ was investigated by ReaxFF MD simulations to computationally model the structural reorganization in SiOC during the annealing phase of sol–gel based silicon carbide synthesis procedure.

Using parameters developed by Newsome et al.,^38^ the structure of the precursor material silicon oxycarbide was examined. At first, the temperature-dependent formation of segregated carbon domains within this glassy material was explored. Three methods for defining atomic neighbors within the solid were successfully implemented, all of which yield comparable results. However, for the investigated system the MV^0.5^ method is deemed the most suitable, because the classification has proven to be reasonably robust with only moderate fluctuations while employing a parametrization that is both clearly motivated and promises at least limited transferability.

Also, it was found that the average carbon nanodomain size can be expressed as an exponential function of temperature in the investigated range from 300 to 1000 K. Nevertheless, additional research into the temperature dependent formation of carbon domains in SiOC could provide further insights. Most notably, the investigation of even higher equilibration temperatures 1000 K < *T* < 1800 K would be of particular interest, in order to close the knowledge gap between the moderatly pronounced formation of nanodomains at ≤1000 K as reported in this work and the formation of segregated carbon phases in the solid with an even higher degree of ordering as reported elsewhere.^21−23,28,29^ However, this research would require a drastic increase of the size of the simulation cell to accurately model the exponentially larger carbon domains that are likely to emerge at even higher temperatures. Also, the investigation of a larger range of compositions and initial configurations would improve the robustness of the developed model.

Besides these structural analyses concerning carbon aggregation, powder X-ray diffraction patterns of SiOC were calculated. These diffractograms are in good agreement with experimental PXRD patterns — both theoretical and experimental patterns show similar features and suggest that SiOC is indeed strongly amorphous. Further, the average size of the carbon nanodomains was determined to exert a minor influence on the PXRD patterns.

## Supporting Information Available

Figures for all *RICn*(*n* = 1–3) systems presenting the temporal evolution of the CND size analyzed with the GC^2.0^, LV^4.35^, and MV^0.5^ algorithms; temperature-dependent equilibrium values of CND size; time-averaged histograms of CC distances and time-averaged PXRD patterns; table of the calculated PXRD reflexes of graphite, 3C-SiC, and SiO_2_; and figure presenting the recorded Raman spectrum of the SiOC sample (PDF)
