# Supplementary material for: Temperature-Dependent Formation of Carbon Nanodomains in Silicon Oxycarbide Glass—A Reactive Force Field MD Study
Source: J Phys Chem C Nanomater Interfaces. 2024 Dec 16;129(1):552–61. doi: 10.1021/acs.jpcc.4c05132 (PMC11726657; doi:10.1021/acs.jpcc.4c05132)
Supplement: Supplementary file 1 — jp4c05132_si_001.pdf [file jp4c05132_si_001.pdf]

Supplementary Material

Temperature-Dependent Formation of Carbon Nanodomains in Silicon Oxycarbide Glass—A Reactive Force Field MD Study

Bernhard M. Kriesche[a], Felix R. S. Purtscher[a], Benedikt E. Hörfarter[a], Teja Stüwe[b], Victoria Greussing[b], Bettina Friedel[c], Engelbert Portenkirchner[b] and Thomas S. Hofer[a*]

[a] Institute of General, Inorganic and Theoretical Chemistry
Center for Chemistry and Biomedicine
University of Innsbruck,
Innrain 80-82, A-6020 Innsbruck, Austria

[b] Institute of Physical Chemistry
University of Innsbruck,
Innrain 52c, A-6020 Innsbruck, Austria

[c] Department 4.5 Applied Radiometry
Physikalisch-Technische Bundesanstalt (PTB),
Bundesallee 100, D-38116 Braunschweig, Germany

*Corresponding author: t.hofer@uibk.ac.at

S1 Results

Figs. S1 to S9 show the temporal evolution of the carbon nanodomain size analyzed with the $GC^{2.0}$, $LV^{4.35}$ and $MV^{0.5}$ algorithms in SiOC/*RICn* (n=1-3) systems. The grey boxes indicate the terminal 5 ns of each simulation, the resulting equilibrium values are indicated by the dashed horizontal lines.

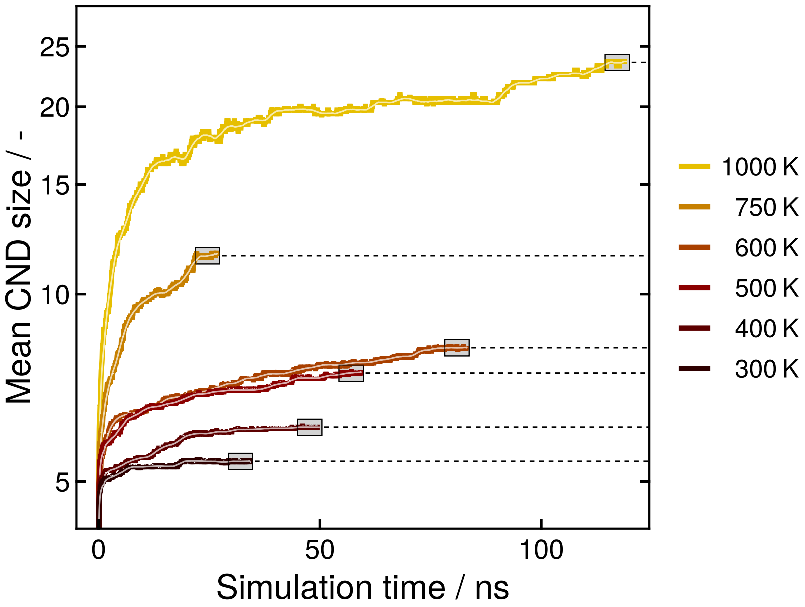

Figure S1: Time evolution of average carbon nanodomain size obtained with the $GC^{2.0}$ algorithm in the *RIC1* system at various temperatures.

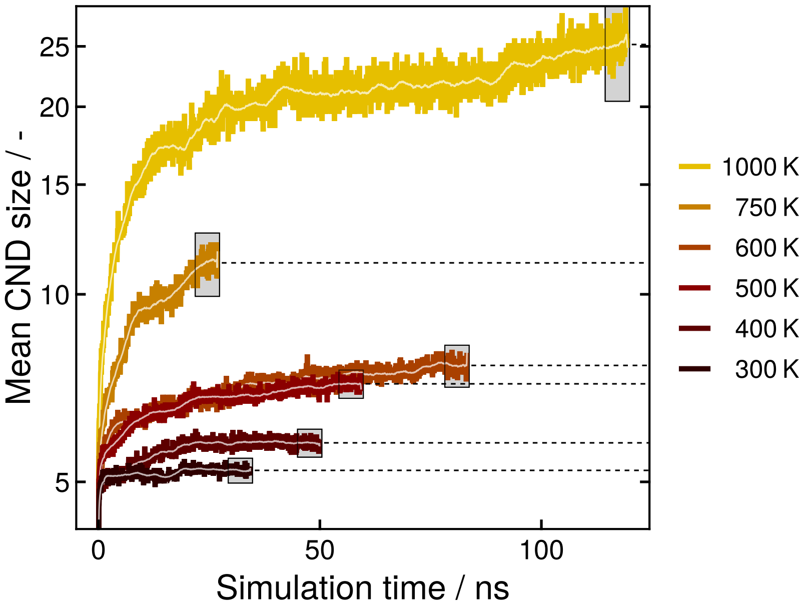

Figure S2: Time evolution of average carbon nanodomain size obtained with the $LV^{4.35}$ algorithm in the *RIC1* system at various temperatures.

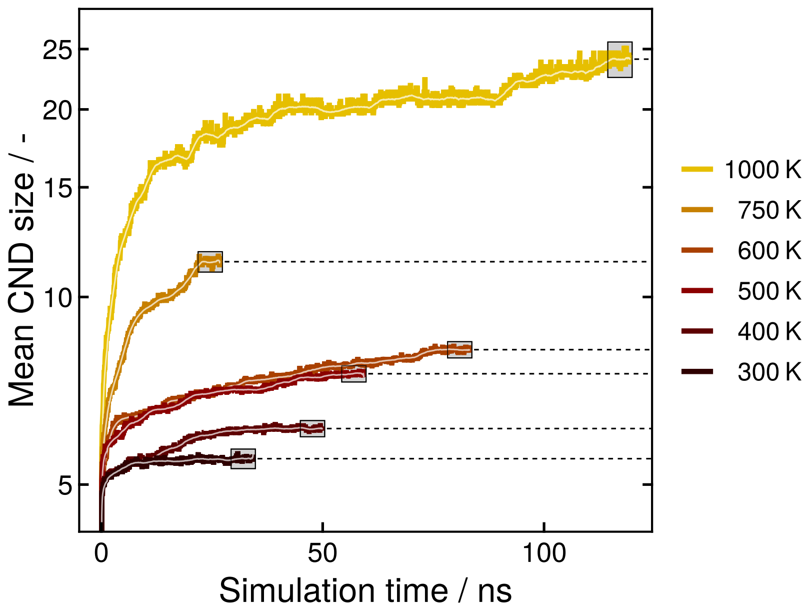

Figure S3: Time evolution of average carbon nanodomain size obtained with the $MV^{0.5}$ algorithm in the *RIC1* system at various temperatures.

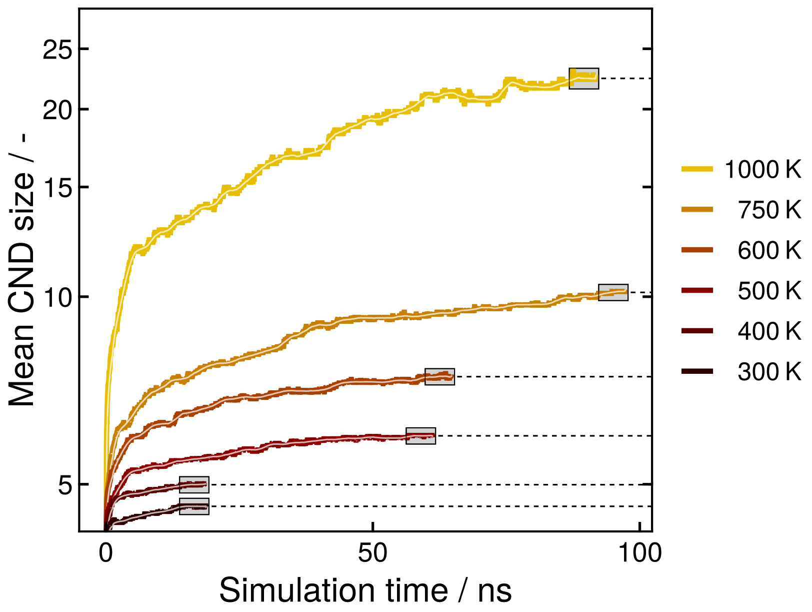

Figure S4: Time evolution of average carbon nanodomain size obtained with the $GC^{2.0}$ algorithm in the *RIC2* system at various temperatures.

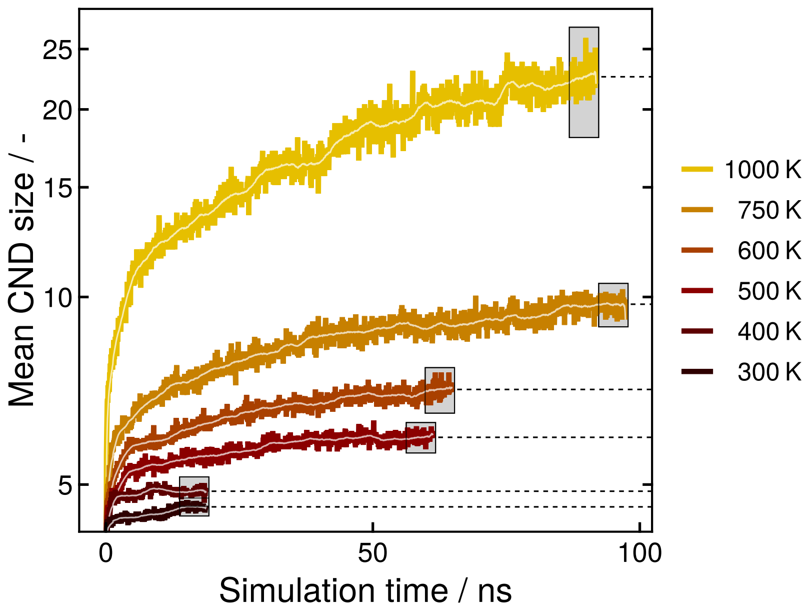

Figure S5: Time evolution of average carbon nanodomain size obtained with the $LV^{4.35}$ algorithm in the *RIC2* system at various temperatures.

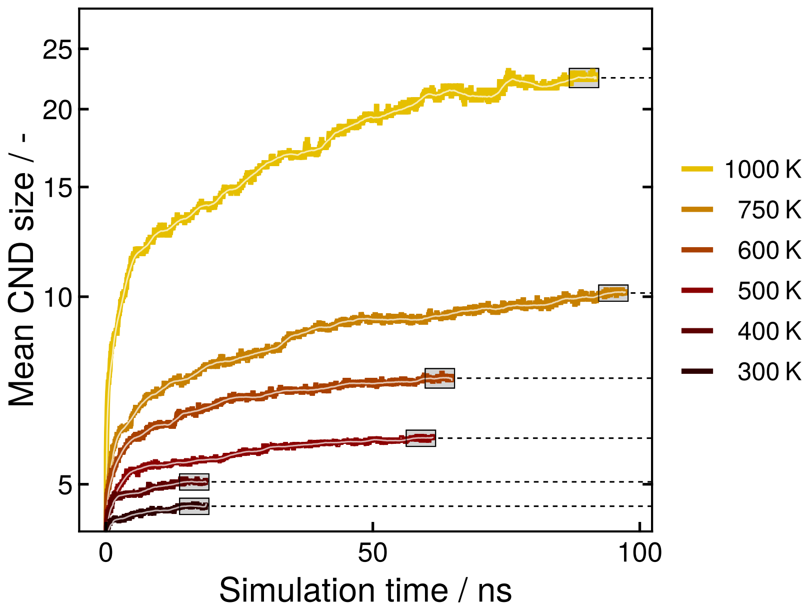

Figure S6: Time evolution of average carbon nanodomain size obtained with the $MV^{0.5}$ algorithm in the *RIC2* system at various temperatures.

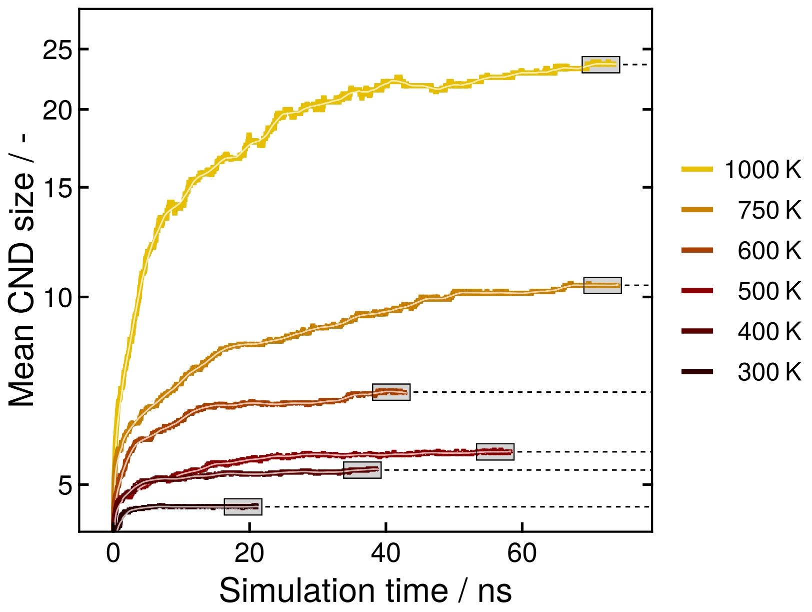

Figure S7: Time evolution of average carbon nanodomain size obtained with the $GC^{2.0}$ algorithm in the *RIC3* system at various temperatures.

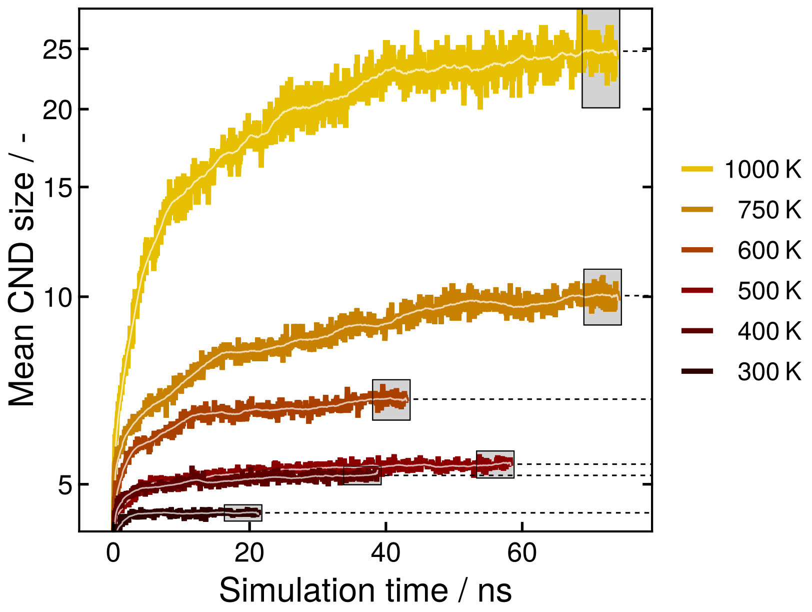

Figure S8: Time evolution of average carbon nanodomain size obtained with the $LV^{4.35}$ algorithm in the *RIC3* system at various temperatures.

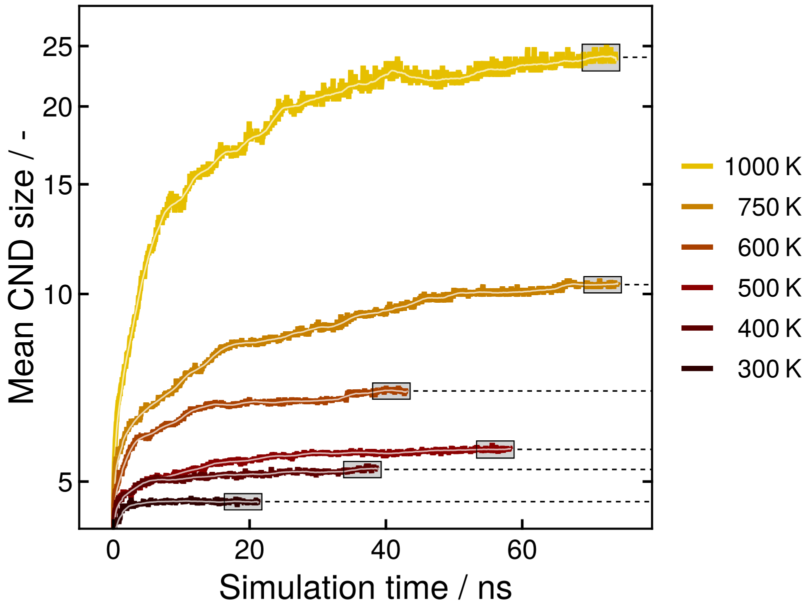

Figure S9: Time evolution of average carbon nanodomain size obtained with the $MV^{0.5}$ algorithm in the *RIC3* system at various temperatures.

Figs. S10 to S12 show the average equilibrium nanodomain size against simulation temperature and fit to an exponential model, obtain with the $GC^{2.0}$, $LV^{4.35}$ and $MV^{0.5}$ algorithms, respectively.

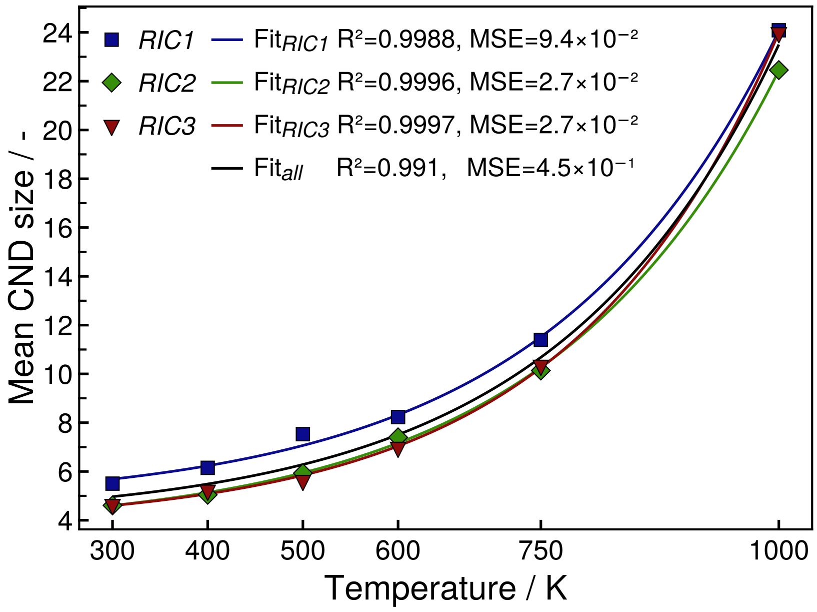

Figure S10: Equilibrium values of average carbon nanodomain size against simulation temperature determined with the $GC^{2.0}$ algorithm and fit to an exponential model (see Eq. 2).

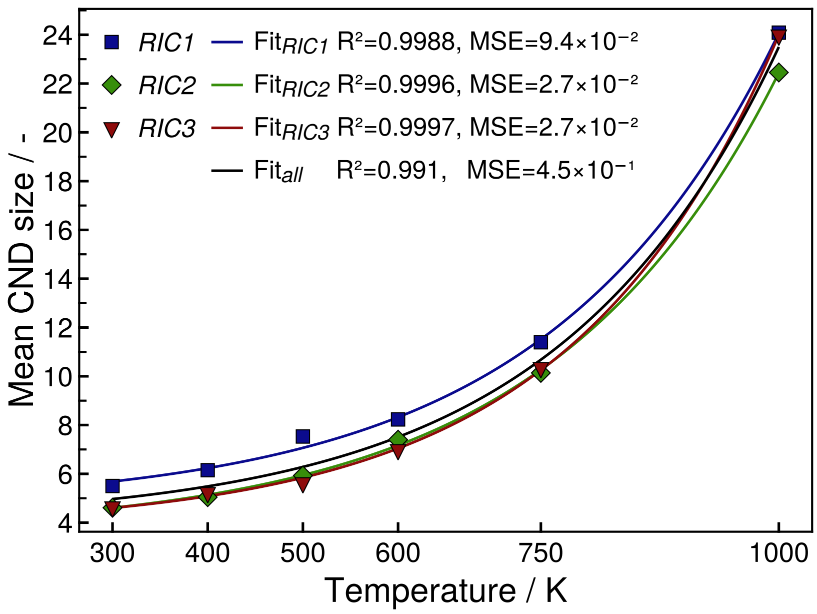

Figure S11: Equilibrium values of average carbon nanodomain size against simulation temperature determined with the $LV^{4.35}$ algorithm and fit to an exponential model (see Eq. 2).

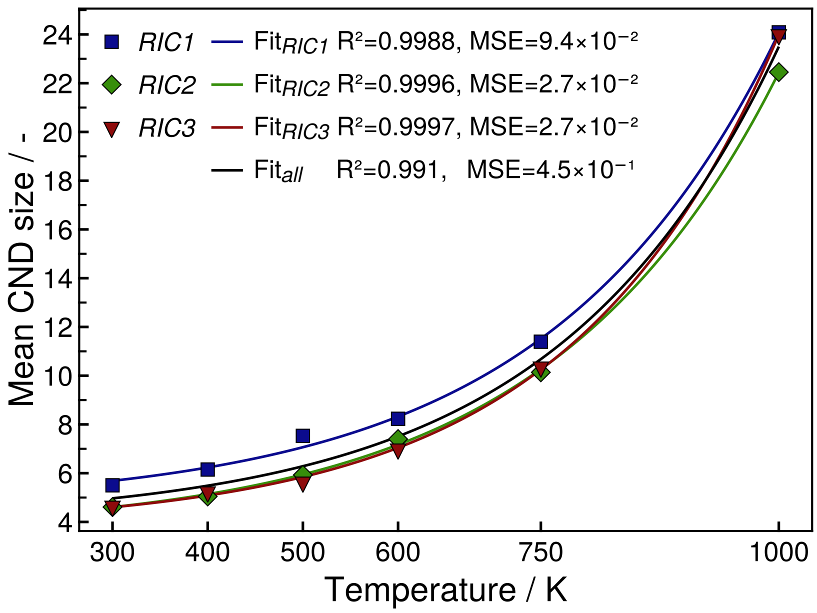

Figure S12: Equilibrium values of average carbon nanodomain size against simulation temperature determined with the $MV^{0.5}$ algorithm and fit to an exponential model (see Eq. 2).

Figs. S13 to S15 show time averaged histograms of carbon-carbon distances in all SiOC/*RICn* (*n*=1-3) systems. Reference values indicated via C−C, C⩦C and C≡C denote the median values of single, aromatic and triple carbon-carbon bond distances extracted from ReaxFF MD simulations of diamond, graphite and γ-graphyne, respectively.
Tab. S1 lists the calculated XRD reflexes of graphite, 3C-SiC and SiO_2 at angles $5° \leq 2\theta \leq 35°$

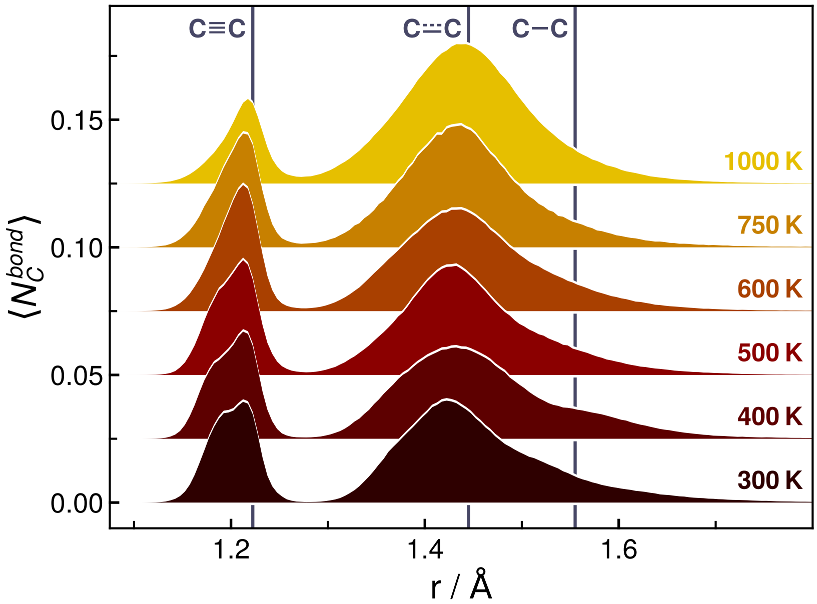

Figure S13: Time averaged histogram of CC distances in SiOC/*RIC1* after equilibration at different temperatures, normalized according to the total number of carbon atoms in the system.

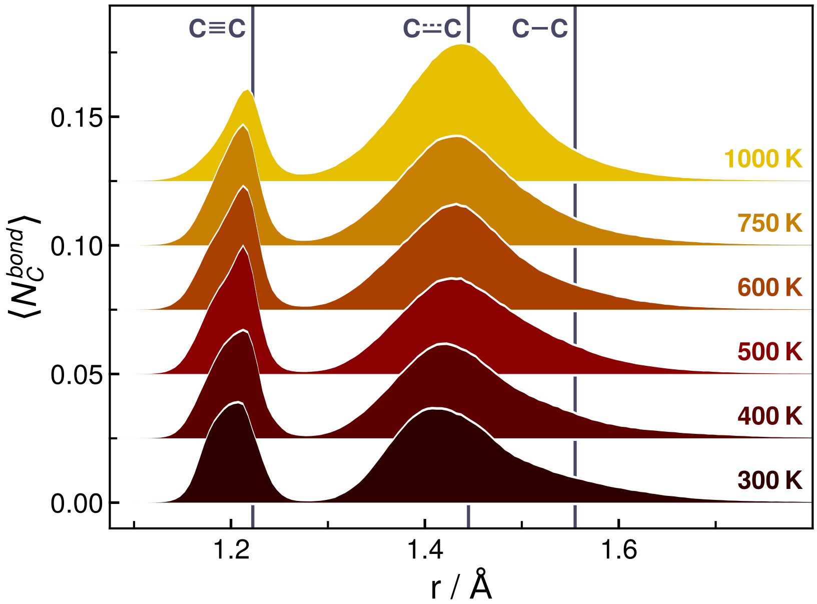

Figure S14: Time averaged histogram of CC distances in SiOC/*RIC2* after equilibration at different temperatures, normalized according to the total number of carbon atoms in the system.

with intensities higher than 1.0% of the strongest reflex, respectively.

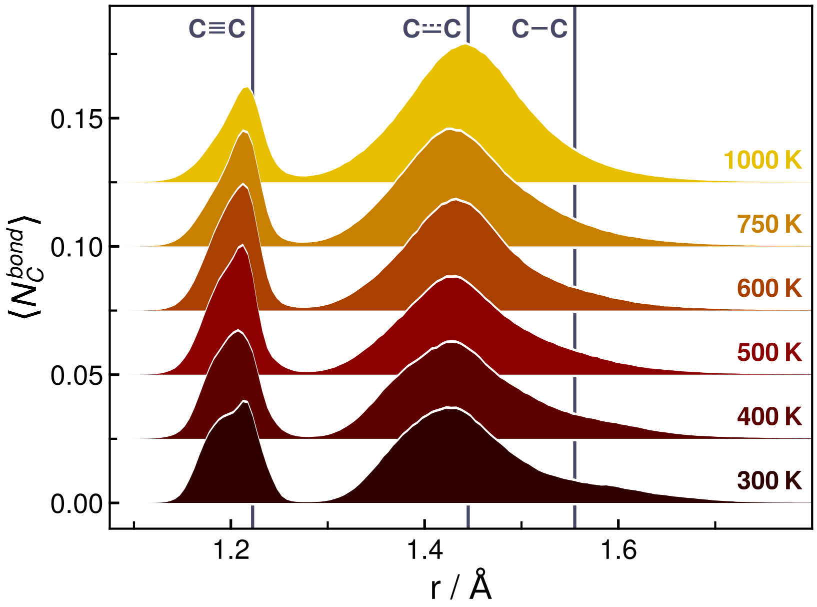

Figure S15: Time averaged histogram of CC distances in SiOC/*RIC3* after equilibration at different temperatures, normalized according to the total number of carbon atoms in the system.

Table S1: Calculated PXRD reflexes of graphite, 3C-SiC and SiO_2 at angles $5° \leq 2\theta \leq 35°$ with intensities higher than 1.0% of the strongest reflex, respectively. The row colors were chosen to reflect the color coding in Fig. 8 in the main manuscript.

2θ / °	I / arb. unit	System
6.18	13.15	graphite
8.25	1.46	graphite
9.64	18.68	SiO_2
10.31	1.10	graphite
12.24	100.00	SiO_2
12.38	100.00	graphite
14.45	1.05	graphite
15.74	100.00	3C-SiC
16.74	3.82	SiO_2
17.92	1.57	SiO_2
18.20	18.6	3C-SiC
18.85	8.75	graphite
19.35	3.72	SiO_2
19.86	23.25	graphite
20.79	4.29	SiO_2
22.60	19.70	SiO_2
22.62	5.30	graphite
24.62	1.18	SiO_2
24.91	5.75	graphite
25.85	53.56	3C-SiC
26.62	6.40	graphite
26.82	8.22	SiO_2
29.22	1.05	SiO_2
29.95	15.11	SiO_2
30.41	42.25	3C-SiC
31.42	2.35	graphite
31.79	6.07	3C-SiC
32.96	8.95	graphite
33.03	1.91	SiO_2
33.86	1.66	SiO_2
34.54	3.75	SiO_2
34.90	3.96	SiO_2

Figs. S16 to S18 compare the measured diffractogram with the calculated PXRD patterns for the SiOC/*RICn* (n=1-3) systems, respectively. Additionally, the reflexes listed in Tab. S1 are also included.

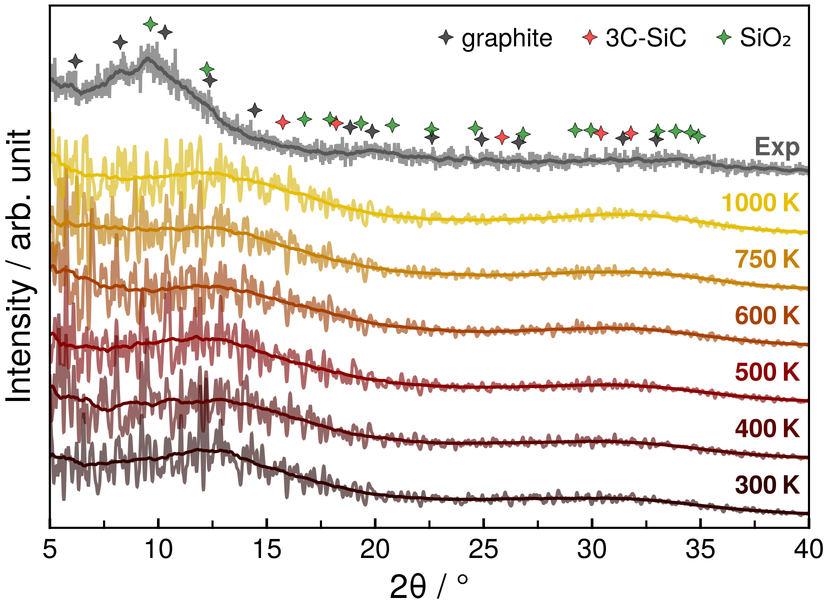

Figure S16: Time-averaged PXRD patterns of SiOC/*RIC1* at different equilibration temperatures compared to the experimentally measured diffractogram. Reflexes obtained for graphite, 3C-SiC and SiO_2 at the respective minimum configuration with intensities being $\geq 0.1\,\%$ of the main reflex are indicated as diamond shaped markers.

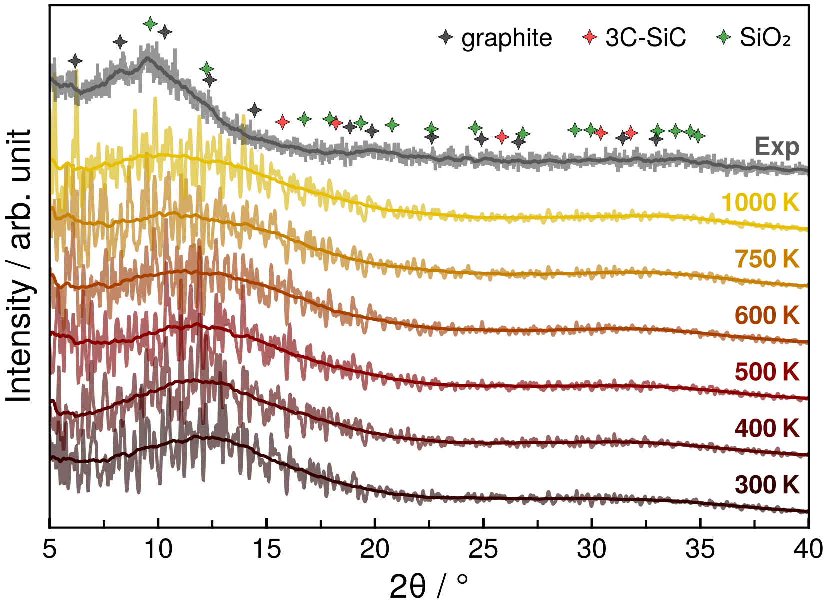

Figure S17: Time-averaged PXRD patterns of SiOC/*RIC2* at different equilibration temperatures compared to the experimentally measured diffractogram. Reflexes obtained for graphite, 3C-SiC and SiO_2 at the respective minimum configuration with intensities being $\geq 0.1\,\%$ of the main reflex are indicated as diamond shaped markers.

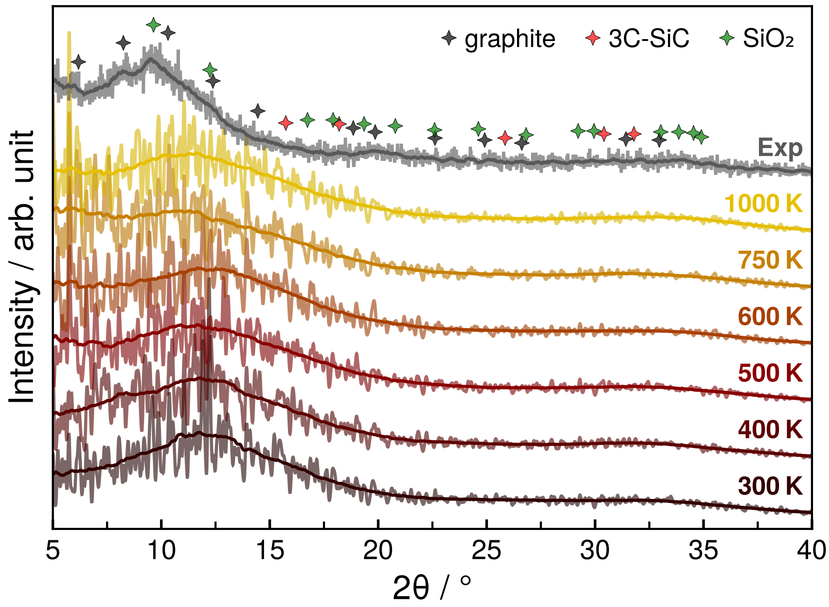

Figure S18: Time-averaged PXRD patterns of SiOC/*RIC3* at different equilibration temperatures compared to the experimentally measured diffractogram. Reflexes obtained for graphite, 3C-SiC and SiO_2 at the respective minimum configuration with intensities being $\geq 0.1\,\%$ of the main reflex are indicated as diamond shaped markers.

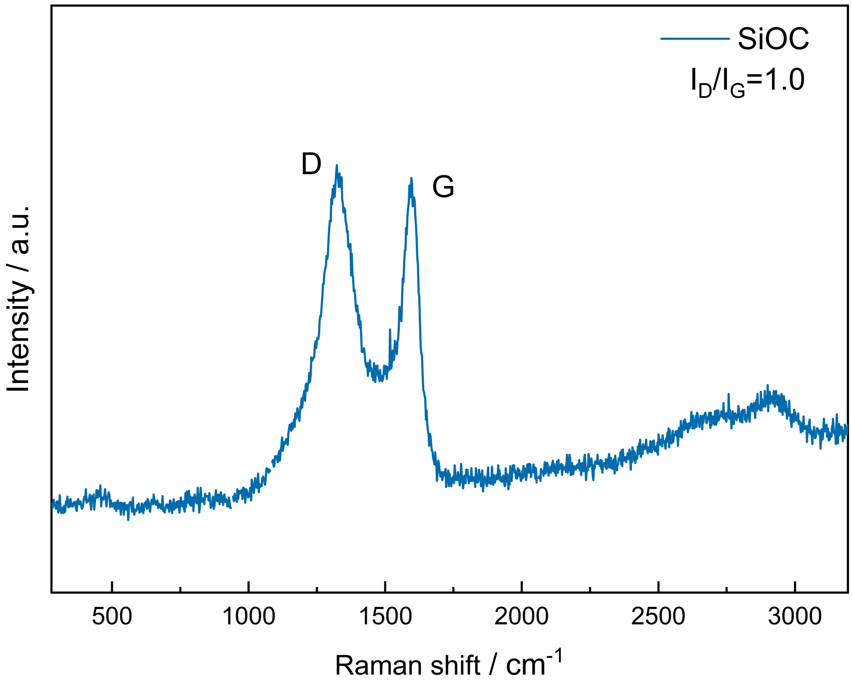

Figure S19: Raman spectrum of SiOC sample used in experimental procedures.
